# Oncogenic Roles of UHRF1 in Cancer

**DOI:** 10.3390/epigenomes8030026

**Published:** 2024-07-01

**Authors:** Ahhyun Kim, Claudia A. Benavente

**Affiliations:** 1 Department of Pharmaceutical Sciences, University of California, Irvine, CA 92697, USA; 2 Department of Developmental and Cell Biology, University of California, Irvine, CA 92697, USA; 3 Chao Family Comprehensive Cancer Center, University of California, Irvine, CA 92697, USA

**Keywords:** UHRF1, cancer, epigenetics

## Abstract

Ubiquitin-like with PHD and RING finger domains 1 (UHRF1) is an essential protein involved in the maintenance of repressive epigenetic marks, ensuring epigenetic stability and fidelity. As an epigenetic regulator, UHRF1 comprises several functional domains (UBL, TTD, PHD, SRA, RING) that are collectively responsible for processes like DNA methylation, histone modification, and DNA repair. UHRF1 is a downstream effector of the RB/E2F pathway, which is nearly universally deregulated in cancer. Under physiological conditions, UHRF1 protein levels are cell cycle-dependent and are post-translationally regulated by proteasomal degradation. Conversely, UHRF1 is overexpressed and serves as an oncogenic driver in multiple cancers. This review focuses on the functional domains of UHRF1, highlighting its key interacting proteins and oncogenic roles in solid tumors including retinoblastoma, osteosarcoma, lung cancer, and breast cancer. Additionally, current therapeutic strategies targeting UHRF1 domains or its interactors are explored, providing an insight on potential clinical applications.

## 1. Introduction

Ubiquitin-like with PHD and RING finger domains 1 (UHRF1) is a critical protein implicated in the regulation of epigenetic landscapes, significantly influencing gene expression patterns within cells [1,2,3]. UHRF1 is a multifunctional protein with a pivotal role in the maintenance of repressive epigenetic marks, thus ensuring epigenetic stability and fidelity. Functioning as both an epigenetic reader and writer, UHRF1 is essential for the processes of DNA methylation and histone modification [4,5]. 

**Figure 1:**
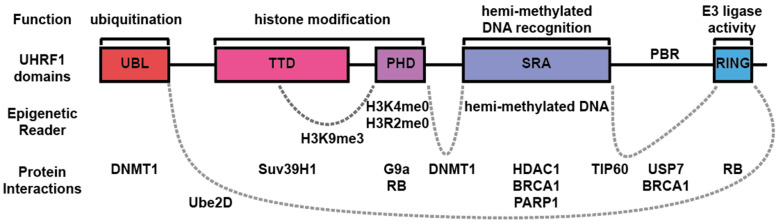
Representation of UHRF1 functional domains and their functions. UHRF1 is a multidomain protein consisting of five domains: ubiquitin-like (UBL) domain, tandem tudor domain (TTD), plant homeodomain (PHD), SET and RING-associated (SRA) domain, and Really Interesting New Gene (RING) domain. These domains are connected by linker regions. Through these domains and linker regions, UHRF1 can function as an epigenetic reader, recognizing histone and DNA modifications including the following: unmodified H3K4 (H3K4me0) and H3R2 (H3R2me0), trimethylated H3K9 (H3K9me3), and hemi-methylated DNA. UHRF1 also interacts with other proteins to exert diverse cellular roles including maintenance of DNA methylation through the binding of DNMT1; maintenance of heterochromatin through binding with Suv39H1, G9a, and HDAC1; DNA damage response through its interaction with TIP60, BRCA1, and PARP1; cell cycle control through binding with RB; and protein degradation through the binding to E2 ligases like Ube2D, which include auto-ubiquitination. Binding to the USP7 deubiquitinase prevents UHRF1 proteasome degradation.

UHRF1 comprises several functional domains that are collectively responsible for its role in gene expression regulation. The ubiquitin-like (UBL) domain facilitates the binding to DNA methyltransferase 1 (DNMT1) [6,7]. The tandem tudor domain (TTD) and plant homeodomain (PHD) are both essential for the recognition of the trimethylated lysine 9 on histone H3 (H3K9me3), a histone mark necessary for DNA methylation [8,9]. The SET and RING-associated (SRA) domain, which recognizes and binds to hemi-methylated DNA, is essential for the maintenance of DNA methylation patterns by recruiting DNMT1 [10,11]. The Really Interesting New Gene domain (RING) is involved in the recruitment of Ube2D for the ubiquitination of histone H3 in coordination with the UBL domain to further influence the chromatin structure and gene expression [6,7,12] (Figure 1).

**Figure 2:**
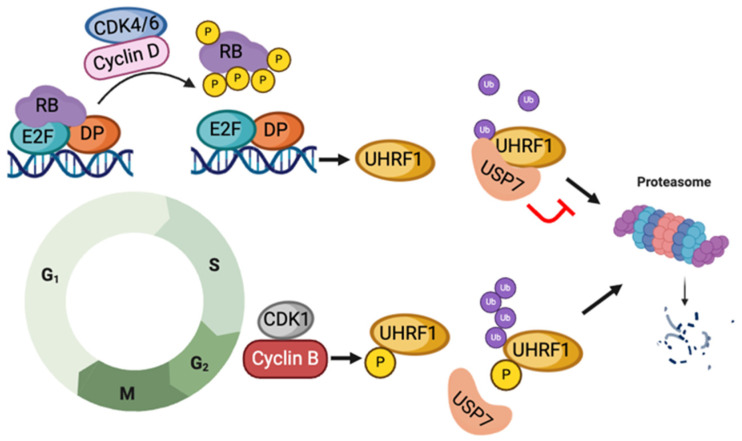
Schematic model of UHRF1 regulation during cell cycle. In proliferating cells, activated cyclin D/CDK complex phosphorylates RB, causing activation of E2F1. Activated E2F1 binds to the promoter of genes necessary for cell cycle progression including UHRF1. Deubiquitinase USP7 interaction with UHRF1 during S phase deubiquitinates UHRF1 and prevents its proteasomal degradation. Activated cyclin B/CDK complex phosphorylates UHRF1 upon completion of S phase, which prevents USP7 binding and leads to UHRF1 degradation by the proteasome.

The regulation of UHRF1 is achieved transcriptionally through the RB/E2F pathway [13,14]. UHRF1 protein levels are post-translationally regulated by proteasome-mediated degradation (Figure 2) [15,16]. Under physiological conditions, UHRF1 expression is primarily observed during the G1/S phase of the cell cycle in actively proliferating cells, with a reduction in expression as cells progress through the M phase. This is further regulated by the phosphorylation of UHRF1 at the Ubiquitin-Specific Protease 7 (USP7)-interacting region, which prevents USP7 binding, leading to UHRF1 proteasomal degradation [15].

In contrast, in cancerous cells, UHRF1 expression is upregulated due to E2F1 deregulation, maintaining high levels of UHRF1 throughout the cell cycle [17]. This overexpression is observed in multiple cancers, including retinoblastoma [13,18], osteosarcoma [14,19], and breast [20,21], lung [22], and pancreatic cancer [23,24]. It is posited that UHRF1 overexpression contributes to tumorigenesis through the silencing of tumor suppressor genes by the aberrant hypermethylation of promoters [25,26]. Concomitantly, as an E3 ubiquitin ligase, UHRF1’s overexpression can also facilitate the degradation of DNMT1, leading to genomic hypomethylation [27].

This review focuses on the functional significance of each UHRF1 domain, their key interactions, and the mechanistic insights into UHRF1’s role as a facilitator of oncogenesis in various solid tumors. Furthermore, we explore the current therapeutic strategies targeting UHRF1 indirectly, offering a perspective on potential clinical implications.

## 2. UHRF1 Functional Domains

### 2.1. UBL Domain

The recent studies unraveling the intricacies of DNMT1 localization through ubiquitinated histone H3 underscore the pivotal role of ubiquitin and UHRF1’s UBL-domain interaction with DNMT1 [6]. The elucidation of the crystal structure showcasing ubiquitin in complex with DNMT1’s Replication Focus Targeting Sequence (RFTS) domain marks a significant advance in understanding the spatial configuration and implications of these molecular interactions [6]. This complex formation induces a structural alteration, notably a bending connector helix between the alpha-helical bundle and the beta-barrel, a deviation from the straight conformation observed when DNMT1 is unbound [6].

Mutational analysis have delineated the critical regions within the RFTS domain necessary for ubiquitin association, revealing that while these regions are indispensable for ubiquitin binding, they are not essential for the interaction with UHRF1’s UBL domain [6]. This finding suggests a nuanced specificity in the molecular interplay involved in DNMT1 regulation. Typically, DNMT1 is self-regulated by the RFTS domain’s occlusion of the catalytic pocket, inhibiting substrate access [28]. However, this autoregulation is modulated upon the UHRF1 UBL domain’s interaction with DNMT1 in the presence of hemi-methylated DNA, enhancing DNMT1’s enzymatic activity compared to ubiquitin interaction alone [6].

Mass spectrometry analyses aimed at understanding the UBL domain’s positioning within UHRF1 have indicated an internal folding pattern, suggesting a compact intramolecular arrangement. Contrary to what one might expect from its structural configuration, functional assays employing a UBL-deleted UHRF1 suggest that this domain does not play a direct role in chromatin association, adding a layer of complexity to the understanding of UHRF1’s function in epigenetic regulation [7].

Further insights into the UBL domain’s role are provided by its involvement in the ubiquitination of histone H3, where it exhibits a preference for binding the E2 enzyme compared to the RING domain, previously believed to be the primary mediator in recruiting ubiquitin-bound E2 to form the ubiquitin ligase E2/E3 complex. The binding of both the UBL domain and ubiquitin at the “backside” of Ube2D forms structurally distinct complexes, underscoring the intricacy of the interactions facilitating enzyme recruitment and substrate modification [12]. These revelations highlight the elaborate regulatory mechanisms controlling DNMT1’s activity and localization, showcasing the UBL domain’s multifaceted role in these processes.

### 2.2. TTD and PHD

Research has elucidated the nuanced roles of UHRF1’s tandem tudor domain (TTD) and plant homeodomain (PHD) in the recognition of histone H3 marks, revealing a sophisticated mechanism of epigenetic regulation. Specifically, the PHD has been shown to recognize the unmodified state of histone H3 while the TTD is responsible for identifying H3 when it is trimethylated on lysine 9 (H3K9me3), a mark commonly associated with gene silencing and heterochromatin formation [29,30]. Together, the TTD and PHD are involved in histone H3K9 methylation initiated by the histone lysine methyltransferases Suv39H1 and G9a [31].

Studies employing X-ray crystallography have demonstrated that both the TTD and PHD are essential for the recognition of H3K9me3. The presence of PHD enhances the interaction between the TTD and the methylated H3, indicating a synergistic action between these two domains in recognizing the epigenetic mark [8]. Intriguingly, fluorescence resonance energy transfer (FRET) assays have observed an increase in the spatial distance between the TTD and PHD upon binding to the H3K9me3 peptide. This suggests a conformational change of the TTD–PHD complex, which intriguingly does not affect UHRF1’s functions including its auto-ubiquitination activity or its affinity for hemi-methylated DNA [8].

An additional layer of complexity in UHRF1’s regulation is provided by the polybasic region (PBR), located between the SRA and RING domains. The PBR engages in an intramolecular interaction with the TTD, resulting in a closed conformation of UHRF1. This interaction hinders the TTD’s ability to bind H3K9me3, thereby regulating UHRF1’s association with chromatin. The transition of UHRF1 to an open conformation, conducive for heterochromatin binding, requires the involvement of interactors like USP7, which interact with the PBR [32].

The specificity of the TTD and PHD’s binding to the H3 tail is underscored by mutational analyses. Alterations to two acidic residues in the PHD (D334A/E335A) result in a complete loss of binding to the H3 tail despite the presence of a functional TTD. Similarly, a mutation in the TTD (Y188A) only weakens the strength of interaction with methylated H3K9 rather than completely abolishing it [9]. These findings underscore the delicate interplay between the TTD and PHD in recognizing specific histone modifications, highlighting their critical roles in the orchestration of epigenetic regulatory processes.

### 2.3. SRA Domain

Structural studies into UHRF1 have elucidated the selective affinity of its SRA domain for hemi-methylated CpG DNA sites, a preference that plays a critical role in the epigenetic regulation mechanism. A detailed crystallographic analysis has revealed how the SRA domain of UHRF1 forms a complex with hemi-methylated DNA [33]. This interaction is strikingly characterized by the outward flipping of the methylcytosine base from the DNA helix within the SRA–DNA complex, a structural peculiarity that facilitates the transfer of methylation marks, underscoring a coordinated effort with DNMT1 for the maintenance of DNA methylation patterns [34]. The specificity of the SRA domain’s binding to hemi-methylated DNA is attributed to key amino acid residues conserved within the domain. Among these, the mutation D469G has been identified to significantly diminish the binding affinity of the SRA domain to the hemi-methylated DNA, highlighting the importance of these conserved residues in the specific recognition process [35].

Further, the SRA domain’s interaction extends to the RFTS domain of DNMT1, a critical interplay necessary for the recruitment of DNMT1 and its subsequent enzymatic activity on hemi-methylated DNA [36]. This interaction is potentiated by UHRF1, which enhances the methylation preference of DNMT1 for hemi-methylated substrates [36]. A competitive binding assay revealed that the direct interaction of RFTS with the SRA domain facilitates the release of the hemi-methylated site from the SRA domain, allowing DNMT1 catalytic access to the substrate [37].

Adjacent to the SRA domain lies a spacer region, located near the C-terminus and enriched with basic residues. Initially, UHRF1 is maintained in a closed conformation wherein the interaction between the SRA spacer and the TTD hampers the domain’s ability to bind to the H3K9me3 mark. However, upon binding to hemi-methylated DNA, UHRF1 undergoes a conformational shift to an open state. In this state, the SRA spacer exhibits a dynamical functionality, toggling between the recognition of hemi-methylated DNA and aiding the recruitment of DNMT1 [5]. This dynamic interplay underlies the intricate role of the SRA spacer in facilitating the versatile roles of UHRF1 in epigenetic regulation and DNA methylation maintenance.

### 2.4. RING Domain

The RING finger domain situated at the C-terminus of UHRF1 is instrumental for providing the protein with its E3 ubiquitin ligase activity. This E3 ligase activity is crucial for UHRF1’s epigenetic processes, particularly by facilitating the recruitment of DNMT1 to DNA replication sites through the ubiquitination of histone H3 on lysine 18 (H3K18) and lysine 23 (H3K23) [38,39]. This targeted ubiquitination serves as a beacon, guiding DNMT1’s function in maintaining DNA methylation patterns during DNA replication, thus ensuring the faithful inheritance of epigenetic information.

Although the UBL domain of UHRF1 exhibits a strong binding for the E2 ubiquitin-conjugating enzyme, specifically Ube2D, it functions in synergy with the RING domain to form connections with the E2 factor [12]. This collaborative interaction underscores the sophisticated coordination between different domains of UHRF1 in executing its role in epigenetic regulation.

A notable element of the RING domain’s function has been identified by studies on thymoquinone, a natural compound known for its potent and selective anti-proliferative and pro-apoptotic effects, particularly pronounced in cancer cells. One study revealed that thymoquinone triggers a rapid auto-ubiquitylation of UHRF1, a process mediated by the RING domain [40]. This auto-ubiquitination marks a significant self-regulatory mechanism, potentially influencing UHRF1 stability and modulating its activity, with implications for cell proliferation and survival.

Moreover, the critical role of the RING domain extends to cellular resilience under stress conditions. Research has elucidated the RING domain’s essential contribution to cell survival, especially when cells are exposed to genotoxic or cytotoxic agents [41]. This capacity for ensuring survival underscores the RING domain’s significance beyond epigenetic regulation, highlighting its involvement in cellular defense mechanisms. Such multifaceted roles of the RING domain, from guiding DNA methylation maintenance to modulating cellular responses to external stresses, exemplify the complex nature of UHRF1’s function in cellular processes and stress adaptation.

In summary, the UHRF1 functional domains collectively contribute to the epigenetic maintenance of DNA methylation. UHRF1 is initially in a closed conformational state in which the TTD is intramolecularly bound to the PBR. Upon the recognition of hemi-methylated DNA, UHRF1 switches to an open conformation, releasing TTD and, with the help of PHD, enhancing its interaction with methylated histone H3 marks. The UBL, in conjunction with the RING domain, preferentially binds to the E2 ubiquitin-conjugating enzyme to promote the ubiquitination of histone H3. The RING domain facilitates DNMT1 recruitment to the hemi-methylated sites through H3 ubiquitination. The SRA domain selectively binds to the hemi-methylated CpG but is released upon the direct interaction of the SRA domain with the RFTS domain of DNMT1, allowing DNMT1 catalytic access for facilitating DNA methylation patterns [28,42].

## 3. UHRF1 Functional Protein Interactions

UHRF1 is a multifaceted protein that plays a pivotal role in the regulation of the epigenome through its diverse functional domains. These domains enable UHRF1 to interact with an extensive network of both protein and non-protein molecules, facilitating a wide range of physiological and pathological processes. This review draws attention to the key protein interactors of UHRF1 that are integral to various critical cellular functions including DNA methylation (DNMT1), deubiquitination (USP7), histone modification (HDAC1, TIP60), and DNA repair (BRCA1, PARP1). The nature of these interactions, whether direct with UHRF1 or through physical associations with UHRF1’s interactors, is crucial for understanding UHRF1’s role in epigenetic regulation and its implications for cellular dynamics (Figure 1).

### 3.1. DNMT1

DNMT1 stands out among the family of DNA methyltransferases due to its preference for methylating cytosines within hemi-methylated CpG dinucleotides during DNA replication. This specificity makes DNMT1 essential in maintaining DNA methylation patterns during cell division, safeguarding the epigenetic heritage and ensuring the continuity of gene expression regulation through generations of cells.

The functional synergy between DNMT1 and UHRF1 is fundamental to this maintenance of DNA methylation patterns. UHRF1, adept at recognizing both hemi-methylated DNA as well as H3K9me2/3 marks, acts as a guiding beacon for DNMT1 at DNA replication forks during the S phase of the cell cycle [43]. For this, UHRF1 targets H3 histones at the replication forks for ubiquitination on lysine 18 (H3K18) or 23 (H3K23). This post-translational modification effectively signals for the recruitment of DNMT1 to the replication fork, enabling methylation maintenance [6].

This intricate interaction between DNMT1 and UHRF1, mediated through DNMT1’s RFTS domain, is essential for DNMT1 function [39]. This collaboration not only serves as a molecular bridge but enables DNMT1 to precisely target hemi-methylated CpG sites of replicating DNA efficiently. This precision is critical for preserving the integrity of epigenetic marks and ensuring the fidelity of gene regulation as cells divide.

Aberrant DNA methylation patterns are associated with diseases, especially cancer. Changes in the expression levels of UHRF1 can disrupt its ability to effectively recruit DNMT1 and maintain DNA methylation patterns. This disruption contributes to the irregular DNA methylation patterns often observed in cancer cells. Such alternations in DNA methylation not only compromise genomic integrity but can also lead to the dysregulation of gene expression, contributing to oncogenesis and cancer progression.

Hence, understanding the molecular intricacies and regulatory mechanisms underpinning the DNMT1–UHRF1 interaction not only provides fundamental insights into the maintenance of epigenetic integrity but also highlights potential therapeutic targets in diseases characterized by epigenetic dysregulation.

### 3.2. USP7

USP7, also known as Herpesvirus-Associated Ubiquitin-Specific Protease (HAUSP), is among the most thoroughly investigated deubiquitinases (DUBs), enzymes responsible for removing ubiquitin from substrate proteins. This enzymatic function is crucial for controlling the stability, activity, and localization of proteins. USP7 stands out due to its implicated oncogenic properties in cancer, capturing significant interest for its role in the regulation and maintenance of cellular processes, including those associated with epigenetic mechanisms. A pivotal aspect of USP7’s function is its involvement in the interaction between DNMT1 and UHRF1. This interaction is fundamental to the regulation of DNA methylation. UHRF1 has been shown to ubiquitinate DNMT1, an action that promotes DNMT1 destabilization. In a pivotal counterbalance, USP7 mediates the deubiquitination of both UHRF1 and DNMT1, rescuing them from proteasomal degradation, thus ensuring their stability and function within the cell [44].

Further elucidating the dynamic partnership between these proteins, research has demonstrated how UHRF1 and USP7 work together to modulate the ubiquitination and subsequent deubiquitination of histone H3. This histone plays a role in creating a conducive chromatin environment for DNMT1 to bind and carry out DNA methylation [45].

The interaction between UHRF1 and USP7 is facilitated by a structural complementarity wherein the UBL1-2 domains of USP7 bind to the PBR of UHRF1 [16]. Notably, the site on the PBR that binds to USP7 overlaps with the TTD of UHRF1. This interaction induces a significant conformational shift in UHRF1, transitioning from a TTD-occluded state to an open state. This conformational change is crucial for the processes of deubiquitination and chromatin binding.

In normal cellular physiology, USP7 prevents excessive DNA methylation by suppressing DNMT1 recruitment. This balance is critical for maintaining genomic stability and preventing aberrant gene silencing that can lead to oncogenic transformation. By modulating the interactions and stability of key players in epigenetic regulation, USP7 plays a crucial role in preserving cellular health and preventing disease progression, particularly in cancer.

### 3.3. HDAC1

Histone deacetylase 1 (HDAC1) plays a pivotal role in gene regulation by removing acetyl groups from lysine residues on histones. This results in chromatin compaction, rendering DNA less accessible for transcription and thus downregulating gene expression.

UHRF1 can form a complex interaction with HDAC1, illustrating another facet of its epigenetic regulation [10]. Specifically, the SRA domain of UHRF1 is responsible for facilitating this interaction [10]. This relationship is further complicated and enriched by the presence of a UHRF1-binding partner, namely UHRF1-binding protein 1, which also interacts with HDAC1, suggesting a more complex network of interactions involving these proteins [10]. Additionally, DNMT1 is involved in this complex, linking DNA methylation maintenance directly with histone modification processes, thereby presenting a concerted effort of various epigenetic modifiers in the regulation of gene expression [46].

A study revealed that targeting UHRF1 alone is insufficient to reactivate epigenetically silenced genes, which are often observed in cancer [47]. Instead, a combination of both the depletion of UHRF1 and the inhibition of HDAC has shown promising results in restoring the expression of silenced tumor suppressor genes. This strategy has been particularly effective in colorectal cancer cell lines characterized by a high CpG island methylator phenotype. The utilization of trichostatin A (TSA), an HDAC inhibitor, in conjunction with UHRF1 depletion, not only reactivated silenced genes but also significantly suppressed cell proliferation and induced cell cycle arrest in the G1 and G2/M phases, highlighting a potent therapeutic avenue.

In essence, the intricate interplay between UHRF1 and HDAC1 highlights the significance of these interactions in gene regulation, particularly in the context of epigenetic control and cancer, and underscores the therapeutic potential of targeting this duo in certain disease scenarios.

### 3.4. TIP60

Tat-interactive protein, 60 kDa (TIP60), is a histone acetyltransferase primarily known for its specificity in acetylating the lysine 5 of histone H2A (H2AK5). This multifaceted protein is involved in numerous important cellular processes including DNA damage response and repair, cell growth, and apoptosis [48,49].

TIP60 is involved in a nuclear complex that includes UHRF1, DNMT1, and HDAC [50]. This assembly underscores the coordinated regulation of histone modifications and DNA methylation, key components of the epigenetic landscape that impacts gene expression, DNA repair mechanisms, and cell death. The role of UHRF1 as a crucial mediator in this context is highlighted by its influence on the acetyltransferase activity of TIP60 towards H2AK5. UHRF1’s presence is a requisite for the acetylation of H2AK5 by TIP60, linking histone acetylation directly with DNMT1-mediated DNA methylation. This connection marks a significant insight into the mechanisms by which epigenetic crosstalk is achieved, regulating the accessibility and integrity of genomic DNA.

Further dissecting the molecular interactions within this complex, the relevance of the zinc finger within the MYST domain of TIP60 comes to light. This domain plays a pivotal role in its interaction with UHRF1, specifically through the SRA and RING domains [51]. This interaction elucidates a direct molecular bridge between TIP60 and the epigenetic control exerted by UHRF1.

Interestingly, TIP60 overexpression results in the downregulation of both UHRF1 and DNMT1, ultimately reducing DNMT1-mediated methylation. Mechanistically, the overexpression of TIP60 leads to UHRF1 auto-ubiquitylation by interfering with the interaction between UHRF1 and USP7 [52]. This perturbation in the protective environment typically afforded to UHRF1 by USP7 hints at the self-regulatory feedback loop wherein TIP60 curtails the epigenetic regulation capabilities of UHRF1 and DNMT1.

In sum, the interactions and consequences of the molecular relationships between TIP60, UHRF1, DNMT1, and HDAC epitomize the intricacy of epigenetic regulation. These dynamics not only influence DNA repair and cell fate decisions but also illuminate potential therapeutic targets for diseases characterized by dysregulated epigenetic landscapes, including various forms of cancer.

### 3.5. BRCA1

Breast cancer susceptibility gene 1 (BRCA1) functions as a tumor suppressor protein that is pivotal in DNA repair processes and the maintenance of genomic stability. Mammalian cells employ two distinct pathways to repair DNA double-strand breaks (DSBs): non-homologous end joining (NHEJ), which operates throughout all cell cycle phases, and homologous recombination (HR), which is specifically active during the S and G2 phases [53].

The direct interaction between BRCA1 and UHRF1 serves as a critical factor in determining the choice of the appropriate DNA repair pathway following DSBs [54]. Upon DNA damage, the phosphorylation event at Ser674 on UHRF1 facilitates its interaction with the BRCT domain of BRCA1, consequently localizing UHRF1 to DSB sites during the S phase. This interaction tips the balance in favor of the HR repair pathway. It is noteworthy that p53-binding protein 1 (53BP1) plays a regulatory role in inhibiting BRCA1-mediated HR in the presence of DSBs, favoring NHEJ through the recruitment of Replication Timing Regulatory Factor 1 (RIF1), a downstream effector in the NHEJ pathway [54].

UHRF1 acts downstream of BRCA1 and is instrumental in removing RIF1 from the DSB site, contributing to the choice of the HR repair pathway [52]. Moreover, UHRF1 has been implicated in regulating the transcription of BRCA1 in sporadic breast cancer. The overexpression of UHRF1 leads to the methylation of the BRCA1 promoter and consequent epigenetic silencing of the BRCA1 gene [55].

This intricate interplay between UHRF1, BRCA1, and auxiliary factors underscores their significant contributions to DNA repair processes and gene regulation, positioning UHRF1 as a key player in the maintenance of genomic integrity and the prevention of diseases, particularly cancer.

### 3.6. PARP1

Poly(ADP-ribose) polymerase 1 (PARP1) is a nuclear protein that plays an essential role in multiple DNA repair pathways. PARP1 acts as a critical modulator of heterochromatin-associated events regulated in part by UHRF1 [56]. PARP1 significantly contributes to the assembly and functional stability of the UHRF1–DNMT1 complex, highlighting its integral role in the maintenance of genomic integrity.

PARP1’s DNA-binding domain preferentially interacts with UHRF1’s SRA domain. The absence of PARP1 weakens the interaction between UHRF1 and DNMT1 without impairing the recruitment of DNMT1 to heterochromatin regions [56]. Furthermore, PARP1 exerts a repressive effect on the E3 ubiquitin ligase activity of UHRF1, particularly in relation to the ubiquitination and subsequent stabilization of DNMT1. This indicates PARP1’s regulatory capacity in modulating protein stability and function within the chromatin context.

Another study revealed PARP1’s predilection for engaging with the methylated form of UHRF1 [57]. This specificity is essential for recruiting UHRF1 to sites of DNA damage, facilitating efficient and accurate repair through the HR pathway [57].

Additionally, the physiological interaction between PARP1 and BRCA1, wherein PARP1 modifies BRCA1 through poly(ADP-ribosyl)ation, corroborates the synergistic action of PARP1 and BRCA1 in mediating HR DNA repair [58]. Consequently, PARP inhibitors have emerged as a focal point in the therapeutic targeting of cancers harboring BRCA1/2 mutations, leveraging the dependency of these cancer cells on PARP1-mediated repair pathways [59].

In summary, PARP1 is intricately involved in various aspects of DNA repair and epigenetic regulation, collaborating with UHRF1 to ensure the integrity and stability of the genome. This orchestrates a finely tuned regulatory network essential for cellular homeostasis and serves as a critical axis for therapeutic interventions in malignancies characterized by compromised DNA repair capacities.

## 4. UHRF1 in Cancers

Currently, no direct evidence links UHRF1 mutations to cancer. However, UHRF1 is often overexpressed in cancers that have inactivated the RB/E2F pathway. These abnormal increases in UHRF1 levels are involved in various disease progression pathways and are associated with a poor clinical outcome, making UHRF1 a promising target for these aggressive forms of cancer.

For instance, in a study in mouse models prone to retinoblastoma, both mRNA and protein levels of UHRF1 were notably high, suggesting its role in tumor initiation and progression [13]. Recent studies in osteosarcoma identified the overexpression of UHRF1 in several human osteosarcoma cell lines, indicating its significance in this cancer type [14,19]. Similarly, in lung cancer, even at early stages, UHRF1 overexpression has significant implications for disease progression and treatment response [22]. In the case of colorectal cancer (CRC), the increased expression of UHRF1 inversely correlates with the expression of the tumor suppressor gene p16^ink4a^, highlighting a significant healthcare burden [60]. Pancreatic cancer, known for its poor survival rates, also displays elevated UHRF1 expression levels, which correlate with lower patient survival rates [23]. Moreover, in medulloblastoma, patients with high UHRF1 expression have significantly shorter overall survival and progression-free survival, reinforcing its prognostic value [61].

The next section underscores UHRF1’s roles across several hallmarks of cancer, including promoting cell proliferation, avoiding cell death, inducing angiogenesis and metastasis, contributing to drug resistance, and the epigenetic silencing of tumor suppressor genes (Table 1).

### 4.1. Sustaining Proliferation and Tumor Growth

UHRF1 has been identified as a significant promoter of cell proliferation and tumor growth in numerous cancers, including retinoblastoma, osteosarcoma, lung cancer, and breast cancer [14,18,20,62,65]. Specifically, in small cell lung cancer (SCLC), UHRF1 promotes SCLC proliferation through its interaction with the yes-associated protein 1 (YAP1), preventing YAP1 ubiquitination and degradation [62]. In non-small cell lung cancer (NSCLC), the expression of long non-coding RNA UHRF1 Protein-Associated Transcript (UPAT) contributes to UHRF1 overexpression, which leads to an increase in cell proliferation and enhances the transition from the G1 to the S phase of the cell cycle [63]. In melanoma, a higher expression of UHRF1 is positively linked with the proliferation marker Ki67, highlighting UHRF1’s role in facilitating melanoma cell division and tumor expansion [64]. On the other hand, UHRF1 downregulation can activate the expression of the tumor suppressor gene p16 and limit CDK4 to the cytoplasm in medulloblastoma cell lines, thereby impeding cell cycle progression [61].

Research further suggests that targeting UHRF1, alongside inhibiting the KRAS pathway and its downstream effector PI3K, could synergistically curb cancer cell growth [65]. Studies in mice with KRAS-driven lung cancer have shown that depleting UHRF1 slows tumor growth. Similarly, in a study, lung cancer patients harboring KRAS mutant tumors with high UHRF1 expression had poorer prognosis [65]. In triple-negative breast cancer (TNBC), a subtype notorious for its poor prognosis and lack of targeted therapies, UHRF1 overexpression reduced the G1 cell population and enhanced tumor growth [20].

These findings portray UHRF1’s key role in cell cycle progression and its contribution to uncontrolled cell proliferation in cancer cells.

### 4.2. Resisting Cell Death

Several studies indicate that cancers overexpressing UHRF1 can evade programmed cell death. For example, when UHRF1 is depleted in retinoblastoma cells, there is an increase in apoptotic markers including cleaved caspase-3 and PARP1 [66]. This suggests a pronounced apoptotic response without impairing DNA damage recognition. Additionally, *UHRF1* silencing has been demonstrated to promote apoptosis by increasing caspase-9 expression [18]. The pro-apoptotic effects facilitated by UHRF1 depletion are mediated through the PI3K/Akt signaling pathway, a key regulator of cell survival and tumorigenesis [18]. Similarly, in TNBC, UHRF1 overexpression inhibits apoptosis [20]. However, UHRF1’s role in apoptosis varies with the cancer type. For example, in a study, while UHRF1 downregulation inhibited tumor growth in hepatocellular carcinoma (HCC) cell lines, it did not trigger apoptosis [67]. Therefore, while the overexpression of UHRF1 can help cancer cells evade apoptosis, the effect of reducing UHRF1 on cell death depends on the type of cancer.

### 4.3. Inducing Angiogenesis and Metastasis

In TNBC, UHRF1 overexpression has enhanced tumor growth through the induction of angiogenesis [20]. Likewise, recent studies on osteosarcoma have found that when UHRF1 is overexpressed, osteosarcoma cells induce angiogenesis and become more invasive, ultimately becoming more metastatic [14,19]. This points to a significant role of UHRF1 in the progression of this malignancy. UHRF1 enhances the invasiveness of osteosarcoma cancer cells by downregulating the tumor suppressor E-cadherin and promoting epithelial–mesenchymal transition (EMT), a process pivotal for cancer cell dissemination [19]. At the mechanistic level, these detrimental effects of UHRF1 overexpression are mediated through multiple pathways, including alterations in exosome production, which likely contribute to intercellular communication favoring tumor progression. Moreover, UHRF1 suppresses the activity of AMP-activated protein kinase (AMPK) and decreases the levels of Semaphorin 3E (SEMA3E), both of which are involved in the regulation of angiogenesis [14]. The suppression of AMPK activity and the consequential decrease in SEMA3E levels collectively promote an angiogenic environment, further supporting tumor growth and the spread of osteosarcoma cells.

### 4.4. Increasing Chemoresistance

Innate or acquired, chemoresistance is often a major challenge as it is liable for most relapses and poor patient survival [71]. One retinoblastoma study showed that the targeted depletion of UHRF1 significantly enhances the sensitivity of retinoblastoma cells to a panel of chemotherapeutic agents including etoposide, camptothecin, and carboplatin [66]. A study on SCLC, characterized by an aggressive course and a poor prognosis, showed that reducing UHRF1 levels increased the chemosensitivity of SCLC cells to cisplatin, a cornerstone in the combination chemotherapy regimen for this type of lung cancer [62]. Another study on KRAS mutant lung adenocarcinoma identified UHRF1 as a critical target for cardiac glycosides, potent DSB repair inhibitors, in sensitizing KRAS mutant lung cancer to chemotherapy [68]. Altogether, these studies show UHRF1 as a prospective target to improve chemosensitivity.

### 4.5. Epigenetic Silencing

The epigenetic silencing of tumor suppressor genes is quite common in cancer development and progression, resulting in the transcriptional shutdown of genes that regulate various cellular processes [72]. In NSCLC, UHRF1 downregulation has been linked to the hypomethylation and subsequent reactivation of crucial tumor suppressor genes including *RASSF1*, *CYGB*, and *CDH13* [69]. In sporadic breast cancer, the silencing mechanism of the *BRCA1* gene is facilitated through the formation of a transcriptional complex comprising UHRF1, HDAC1, DNMT1, and G9a, leading to the methylation of the *BRCA1* promoter and consequent inhibition of BRCA1 expression [55]. Additionally, the avenues for modulating UHRF1’s influence on breast cancer progression extend to its downstream genes. For instance, the inhibition of cell proliferation due to UHRF1 depletion can be reversed by silencing its downstream gene, Krüppel-like factor 17 (KLF17) [21]. One study on prostate cancer showed that UHRF1 overexpression is characterized by its affinity for the promoter of silenced genes, where it modulates histone H3K9 methylation via the histone methyltransferase EZH2 [70]. This epigenetic regulation mechanism implicates UHRF1 in the maintenance of gene expression patterns conducive to prostate cancer progression.

Collectively, these insights into UHRF1’s involvement across various cancers emphasize its central roles in modulating tumor growth, cell cycle dynamics, and epigenetic landscapes. The consistent association between UHRF1 overexpression and adverse outcomes across multiple malignancies underscores its value as a universal target for innovative cancer therapies. These findings encourage the pursuit of UHRF1-focused research to develop targeted interventions that could potentially ameliorate prognosis and enhance the efficacy of existing treatment modalities in diverse oncological settings.

## 5. Current Potential UHRF1 Therapeutics

UHRF1 has drawn considerable attention as a therapeutic target due to its significant role in cancer pathogenesis. Despite its potential, the development of UHRF1-specific inhibitors is still in its early stages, facing hurdles due to the protein’s complex structure and multifunctionality. Current research focuses on targeting different domains of UHRF1 and exploring alternative inhibition strategies, aiming to attenuate UHRF1’s oncogenic functions (Table 2).

### 5.1. Targeting UHRF1 Domains

Efforts to disrupt UHRF1’s interaction with key epigenetic markers have led to attempts at creating small-molecule antagonists targeting specific UHRF1 domains like the TTD, PHD, and SRA domain. One study showed that the disruption of either the PHD or SRA domain of UHRF1 impairs its oncogenic properties in CRC, including cell proliferation, anchorage-dependent growth, and tumor metastatic capacity [81]. Thus, much work remains to be done regarding the tissue-specific roles of each domain’s unique contributions to UHRF1’s function as an epigenetic regulator in different organs to better understand its distinct therapeutic opportunities.

TTD: A small-molecule antagonist, NV01, has shown potential by binding to the UHRF1 H3K9me3 binding pocket at a K_d_ value of 5 µM, yet improvements in efficacy are still needed [73]. In a study, another strategy employed fragment-based ligand discovery to target the TTD [74]. Through the screening of a 2300-member fragment library, 2,4-lutidine was identified as a moderately potent hit against the TTD. NMR and X-ray crystallography analyses revealed that 2,4-lutidine binds to crucial sites on the TTD known to be involved in binding the H3K9me3 peptide and the PBR. Since 2,4-lutidine is relatively small and cannot block UHRF1’s intramolecular interaction, a lead optimization strategy involving fragment linking may result in more potent compounds.

PHD: Small molecules targeting the PHD have been identified, exhibiting a more potent disruption of histone interactions than those targeting the TTD [75]. Compound MLD3-5 is particularly notable for its effectiveness at disrupting histone–PHD interaction at a low micromolar range.

SRA Domain: Inhibitors focusing on the SRA domain, such as NSC232003, have shown promise in reducing DNMT1 and UHRF1 interactions, which are pivotal in DNA methylation and gene silencing processes [76]. Interestingly, anthracycline derivatives, including mitoxantrone, idarubicin and doxorubicin, have shown the ability to block the interaction between UHRF1 and hemi-methylated DNA [77].

### 5.2. Alternative Therapeutic Approaches

Besides domain-specific targeting, recent studies have explored innovative approaches that result in decreased UHRF1 levels.

Combination Therapies: The synergistic application of PARP and HDAC inhibitors has demonstrated enhanced efficacy in prostate cancer models, and this has been attributed to the depletion of UHRF1 [78].

Protein Degradation: In a study, AKT phosphorylation inhibitor MK2206 was shown to induce the degradation of UHRF1 [79]. This degradation occurred through the inhibition of UHRF1 phosphorylation induced by AKT1, reducing UHRF1’s interaction with USP7 and promoting its interaction with the E3 ubiquitin ligase BTRC. Similarly, HSP90 inhibitors 17-AAG and 17-DMAG effectively decreased the endogenous levels of UHRF1 in a time- and dose-dependent manner [80]. A reduction in UHRF1 protein levels upon treatment with an HSP90 inhibitor occurred through the ubiquitin-dependent proteasomal degradation pathway.

Altogether, the current potential therapeutics directly or indirectly targeting UHRF1 provide a novel therapeutic avenue for the treatment of solid cancers with aberrant UHRF1 expression, with a focus on the oncogenic drivers of the epigenetic landscape. Aside from the need for a more specific UHRF1 inhibitor, both the combination therapies as well as targeting UHRF1 protein degradation present promising therapeutic outcomes, although translational studies have yet to be explored.

## 6. Conclusions and Future Directions

UHRF1 is an essential epigenetic regulator influencing DNA methylation and histone modifications. Its overexpression can lead to global DNA hypermethylation and the consequent silencing of tumor suppressor genes. UHRF1 is often overabundant in cancer cells, contributing to an environment conducive to uncontrolled proliferation. These characteristics propose UHRF1 as not only a potential biomarker for pathological states but also a promising target for therapeutic intervention in cancer.

This review has navigated the intricate architecture and function of UHRF1, shedding light on the unique contributions of its five domains: the UBL domain, TTD, PHD, SRA domain, and RING domain. These domains collectively contribute to the proper maintenance of DNA methylation and the integrity of the histone code, foundational elements of epigenetic regulation. Highlighting the collaborative role of UHRF1 within the DNMT1–UHRF1 complex and its interactions with key regulators including USP7, HDAC1, and TIP60, we have underscored the multifaceted nature of UHRF1 in maintaining genomic stability. UHRF1’s implication in the progression and prognosis of various solid tumors, including osteosarcoma, lung cancer, and breast cancer, has underscored its pathological significance. The association of UHRF1 overexpression with poor clinical outcomes accentuates the need for innovative strategies to modulate its activity.

While the journey towards the development of UHRF1-specific inhibitors progresses, challenges remain. Current endeavors, including inhibitors targeting UHRF1 domains and alternative approaches focusing on protein degradation and DNA damage repair, pave the way for future advancements. The nuanced understanding of UHRF1’s role across different cancers posits a roadmap for developing targeted therapies that could revolutionize cancer treatment.

The potential of microRNAs (miRNAs) in regulating UHRF1 post-transcriptionally offers an additional layer of complexity and opportunity [82,83,84,85]. As our comprehension of UHRF1’s regulatory networks expands, strategies leveraging miRNAs for therapeutic intervention may become increasingly viable.

In sum, UHRF1’s pivotal role in cancer epigenetics propels it to the forefront of therapeutic targeting. Anticipated advances in the understanding of UHRF1’s structural and functional dynamics are key to unlocking novel therapeutic avenues. As research continues to expand our understanding of UHRF1 and its role in cancer, novel therapeutic strategies are likely to emerge.

## Figures and Tables

**Table 1:** UHRF1’s roles in cancers.

Role in Cancer	Cancer	UHRF1	Reference
Sustaining proliferation and tumor growth	SCLC	YAP1 stability promotes proliferation	[62]
	NSCLC	Increases proliferation, enhances G1/S transition	[63]
	Melanoma	Elevates levels of Ki67, facilitates cell division	[64]
	Medulloblastoma	UHRF1 downregulation activates p16 and limits CDK4	[61]
	KRAS-driven lung cancer	Associated with tumor growth and poor prognosis	[65]
	TNBC	Reduces G1 cell population, enhances tumor growth	[20]
Resisting cell death	Retinoblastoma	UHRF1 depletion increases apoptotic markers	[66]
	TNBC	Inhibits apoptosis	[20]
	HCC	UHRF1 downregulation does not trigger apoptosis	[67]
Inducing angiogenesis and metastasis	Osteosarcoma	Downregulates E-cadherin and promotes EMT	[19]
		Suppresses AMPK and decreases SEMA3E	[14]
Increasing chemoresistance	Retinoblastoma	UHRF1 depletion increases sensitivity to etoposide, etc.	[66]
	SCLC	UHRF1 reduction increases cisplatin chemosensitivity	[62]
	KRAS mutant lung cancer	Target for cardiac glycosides to increase sensitivity	[68]
Epigenetic silencing	NSCLC	UHRF1 downregulation is linked to reactivation of TSGs	[69]
	Breast cancer	Transcriptional complex silences *BRCA1* gene	[55]
	Prostate cancer	Modulates H3K9 methylation via EZH2	[70]

**Table 2:** Current potential UHRF1 therapeutics.

Compound	Target	Function	Reference
NV01	UHRF1 TTD–PHD	Bind H3K9me3	[73]
2,4-lutidine	UHRF1 TTD	Bind critical site involved in H3K9me3 and PBR	[74]
MLD3-5	UHRF1 PHD	Disrupt histone-TTD interaction	[75]
NSC232003	UHRF1 SRA	Target 5-methylcytosine; reduce DNMT1–UHRF1 interaction	[76]
Mitoxantrone, idarubicin	UHRF1 SRA	Inhibit UHRF1-hemi-methylated DNA interaction	[77]
Veliparib with SAHA	BRCA1	Decrease cell viability; induce apoptosis and DNA damage; decrease in BRCA1, which degrades UHRF1	[78]
MK2206	AKT1/USP7	Inhibit AKT1 and induce phosphorylated UHRF1; reduce UHRF1–USP7 binding	[79]
17-AAG/17-DMAG	HSP90	Degrade UHRF1 via ubiquitin–proteasome system	[80]

## Data Availability

Not applicable.
